# Language: UG or Not to Be, That Is the Question

**DOI:** 10.1371/journal.pbio.1002063

**Published:** 2015-02-13

**Authors:** Johan J. Bolhuis, Ian Tattersall, Noam Chomsky, Robert C. Berwick

**Affiliations:** 1 Cognitive Neurobiology and Helmholtz Institute, Departments of Psychology and Biology, Utrecht University, Utrecht, The Netherlands; 2 Department of Zoology and Sidney Sussex College, University of Cambridge, Cambridge, United Kingdom; 3 Division of Anthropology, American Museum of Natural History, New York, New York, United States of America; 4 Department of Linguistics and Philosophy, MIT, Cambridge, Massachusetts, United States of America; 5 Department of Electrical Engineering & Computer Science and Brain and Cognitive Sciences, MIT, Cambridge, Massachusetts, United States of America

## Abstract

Language is not the same as speech or communication; rather, it is a computational cognitive system. It has appeared very recently, consistent with a minimalist view of language’s hierarchical syntactic structure.

Lieberman’s commentary [1] nicely illustrates our original argument [2] that analysis of language evolution is complicated by a lack of agreement about the concepts of both “language” and “evolution.” Here we address each in turn. First, Lieberman argues against the existence of a language faculty in the sense in which we defined it: a domain- and species-specific computational cognitive system that can generate arbitrarily complex hierarchical syntactic structure. In contrast, Lieberman defines language functionally, as a means of “communication,” with human speech as a “key attribute.” However, as we originally argued [2], while externalized language may be used for communication, the two cannot be equated. Language is a computational operation occurring in the mind of an individual, independent of its possible communicative use, while speech is one possible externalization of language (among others such as sign) and is not an essential aspect of it. Lieberman’s arguments are a prime example of fallaciously confounding the function(s) of a trait with its mechanism [3,4].

According to Lieberman [1], language is essentially spoken communication, with a hierarchical structure shared with other motor abilities such as dancing. In this view, the rules for hierarchically organizing lexical items are acquired through associative motor learning, just as humans learn how to walk or use cutlery. This contrasts with our view [2,5] that children can acquire any language because they possess *universal grammar* (UG) [5–8]. Lieberman argues against the basic operation we call “*merge*” because, he claims, different languages assemble words differently. In his view, apparently, hierarchical syntactic structure is achieved differently in different languages. In contrast, the Strong Minimalist Thesis (SMT) holds that the basic operation of *merge* is sufficient to achieve hierarchically structured sentences in any language [5–8]. In our essay [2] we argue that “crucially, *merge* can apply to the results of its own output,” and we show that this recursive feature leads to the potentially unbounded hierarchical expressions characteristic of human language, each of which is systematically interpreted at the conceptual-intentional interface, that is, internal to the mind-brain, not just externalized as speech. Emphasis on speech and the vocal tract is beside the point; as Lenneberg [9] showed nearly 50 years ago, signers acquire and use sign just as speakers do. Many empirical examples illustrate that this sort of structure enters into the internal mental computations of language, both in English and across languages (e.g., [10]). Thus, hierarchical, not linear, syntactic structure determines that “he” and “John” cannot be the same person in “He said John ordered sushi” but can be the same in “While he was holding pasta, John ordered sushi.” The relevant constraint is not the linear order of “he” and “John,” i.e., that a pronoun can be identified with a name only if it appears after the name in left-to-right order. Rather, it is whether a pronoun bears a particular hierarchical structural relationship to a name. Furthermore, precisely the same hierarchical constraint operates in many seemingly distinct linguistic phenomena across all languages that have been seriously studied: the way questions are formed, the interpretation of disjunction, the licensing of negative items, and the “binding” between names and pronouns. This confluence of otherwise disparate constraints is readily explained by positing hierarchical *merge*, as we would expect from any scientific theory. But it remains a puzzle if one assumes only a linear association of the sort Lieberman suggests. Furthermore, a hierarchical composition operator lies at the heart of most contemporary linguistic theories: lexical-functional grammar, head-driven phrase structure grammar, combinatory categorial grammar, tree-adjoining grammar, and so forth. These all contain *merge*, which simply identifies this common property of syntactic hierarchical composition that appears in all such theories, the core of human language computation.

Lieberman also equates “Darwinian processes” with gradual natural selection. This oversimplification does not square with the episodic pattern empirically observed in human biological and technological evolution [11]. Nor is his speculation that Neanderthals had “simple syntax” supported by any evidence from nonhuman primates [5,12]. Furthermore, his assertion that transmitting Levallois stone tool—making skills necessitates language is contradicted by experiment [13]. In contrast, the typical pattern of stasis in stone tool technology before modern *Homo sapiens* emerged argues for a lack of language until very recently [11,14], while the modern vocal tract was acquired before we have any substantial reason to suppose it was recruited for speech. With his emphasis on speech and motor learning, Lieberman naturally puts great weight on the role of *FOXP2* in neural circuits involved in motor control. But to single out just one gene—a transcription factor at that—is odd [2]. The point (that we make in our essay) is, though, that speech is not the same as language. The relationship between genes and the computational and cognitive phenotype we call language is surely complex and runs through a number of intermediate steps, including those involving neurons and brain circuits. *FOXP2* mutations or knockouts may have effects on motor systems, including vocalizations, in a range of animals [15]. But none of this speaks to the computational heart of syntax, *merge*; rather, it affects the “input-output” system, not the central processor. Of course, as we argued, it is quite conceivable that this computational system could have been linked to “possibly preexisting perceptual and motor mechanisms” [2], while mutations in both *FOXP2* and other genes may have played a role in the evolution of those mechanisms. But this is simply preadaptation of an input-output substrate, as commonly found throughout evolution.

Thus, we maintain our key argument [2] that the paleoanthropological evidence suggesting that the faculty of language emerged some 70,000–100,000 years ago is entirely consistent with the simple way in which human language syntax is defined by the Strong Minimalist Thesis.
